# The Glenohumeral Ligaments of the Anterior Aspect of the Shoulder: Anatomical Patterning and Morphometry

**DOI:** 10.3390/jfmk11020209

**Published:** 2026-05-26

**Authors:** Emilio González-Arnay, Artimes García-Parra, Isabel Pérez-Santos, Marye Merce Méndez-Ojeda, Elena Bañón-Boulet, Pablo Díaz-Rojas, Lidia Real-Yanes, Noé Liria-Martín, Marta Rodríguez-Padrón, Mario Fajardo-Pérez

**Affiliations:** 1 Department of Basic Medical Sciences, Division of Human Anatomy and Embryology, University of La Laguna, 38200 Canary Islands, Spain; artimes23@gmail.com (A.G.-P.); pdiaroja@ull.edu.es (P.D.-R.); alu0101138619@ull.edu.es (L.R.-Y.);; 2 Department of Anatomy, Histology and Neuroscience, Autonomous University of Madrid, 28049 Madrid, Spain; 3 MundoFisio (Private Practice), Frontera-El Hierro, 38911 Canary Islands, Spain; 4 Division of Traumatology and Orthopedic Surgery, Canary Islands University Hospital, 38320 Canary Islands, Spain; dramaryemendez@gmail.com; 5 Division of Surgery, University Hospital of Burgos, 09006 Burgos, Castilla y León, Spain; elenabanonboulet@gmail.com; 6 Division of Pathology, Canary Islands University Hospital, 38320 Canary Islands, Spain; nlm7589@gmail.com; 7 Technical Department, Canarian Network of Pathology, 38206 Canary Islands, Spain; 8 Ultradissection (Private Practice), 28049 Madrid, Spain; mfajardoperez@yahoo.com

**Keywords:** rotator cuff, shoulder, glenohumeral, anatomy, ligament

## Abstract

**Background**: The glenohumeral ligaments are key stabilizers of the glenohumeral joint. Three distinct fascicles are usually described from the anterior surface of the shoulder joint: a superior glenohumeral ligament (SGHL); a middle glenohumeral ligament (MGHL); and an anteroinferior glenohumeral ligament (AIGHL). A remarkable interindividual variation has been reported, and there are few data about the patterns of insertion of these ligaments. A recent study provided a much-needed insight into the quantitative details of the glenohumeral ligament insertions. However, there is a lack of studies describing linear measurements (closer to real-life surgical anatomy) of glenohumeral ligamentous insertions according to their pattern. **Methods**: Hereby, we present a Thiel-based anatomical study describing proximal (glenoid) insertions of glenohumeral ligaments in 39 specimens from volunteer body donors to the Applied Anatomy Laboratory at the Autonomous University of Madrid. **Results**: Only 30.8% of cases showed a canonical pattern of SGHL, MGHL, and AIGHL, with scapular insertions ranging from 0.28 to 1.58 cm for SGHL, 0.1–3.6 cm for MGHL, and 0.45–2.05 cm for AIGHL, frequently mixed between the labrum and the osseous edge of the glenoid surface. Most cases show a single glenohumeral ligament inserted, usually in the labrum. A wide range of patterns regarding the number of insertions and their labral or osseous nature is present in our sample. **Conclusions**: Overall, there are three different patterns of glenohumeral ligaments in the anterior aspect of the shoulder joint, with the canonical pattern (three ligaments) represented in less than half of the cases. The morphometric study of the glenohumeral ligaments should consider their pattern of distribution. Also, insertions vary between the labrum and the scapular osseous articular surface.

## 1. Introduction

**Figure 1:**
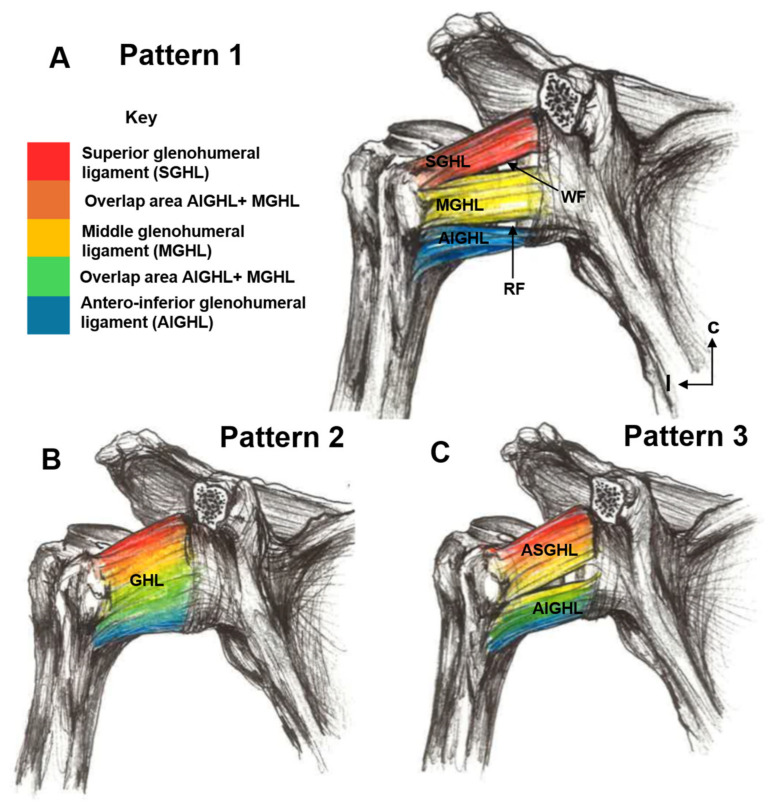
GHLs macroscopic pattern from an anterior view after detachment of the SSM. (**A**): Canonical pattern 1 found in 30.8% of specimens, with three GHLs. (**B**): In 38.8% of specimens GHLs formed a single complex and borders between SGHL/MGHL/AIGHL were not identifiable (pattern 2). (**C**): In 30.8% of specimens, only two ligaments were found, with the presence of a wide ligament partially inserted from 2 o’clock to 4 o’clock being almost a constant (pattern 3); this pattern represents a re-organization of the canonical pattern where the wider fascicle is formed by the amalgamation of the SGHL and the MGHL (anterosuperior glenohumeral ligament, ASGHL) or the amalgamation of MGHL and AIGHL, leaving the remaining fascicle isolated and mirroring its canonical form.

The glenohumeral ligaments are key stabilizers of the glenohumeral joint. First described in the 19th century [1,2,3,4,5], there is no consensus about their detailed anatomy [2,3,4]. Overall, three distinct fascicles (Figure 1A) are usually described from the anterior surface of the shoulder joint: a superior glenohumeral ligament (SGHL) that acts as a stabilizer in adduction and external rotation of the arm [5]; a middle glenohumeral ligament (MGHL) that stabilizes the shoulder in abduction and external rotation [6,7]; and an anteroinferior glenohumeral ligament (AIGHL) that is a pure anterior stabilizer [8]. Furthermore, a posteroinferior glenohumeral ligamentous complex (including PIGHL, which is the posterior band of the AIGHL itself) and a coracohumeral ligament (CHL), which are not readily distinguishable from the anterior surface of the shoulder joint, do contribute to the vertical and posterior stabilization of the shoulder joint. The superior glenohumeral ligament (SGHL) has an origin at the tip of the glenoid cavity and/or from the supraglenoid tubercle, although there are authors that find additional attachments (see Fox et al., 2021 [9] for a comprehensive review and also [10,11,12]); in turn, MGHL originates in the supraglenoid tubercle and the glenoid neck immediately caudal to the insertion of the SGHL. The AIGHL is classically described as one of three fascicles on a broader complex that is almost continuously attached to the caudal half of the osseous labrum [9].

A remarkable interindividual variation has been reported, both from an extraarticular [2,4] and an intraarticular point of view [13,14]. Moreover, there is a lack of universal agreement regarding terminology and quantitative measures of ligament attachments. A recent study [3] provided much-needed insight into the quantitative details of glenohumeral ligament insertions. However, there is a lack of studies describing linear measurements (which are indeed more approximate to real-life surgical anatomy, where surgeons are confronted with a front or rear view of inserted ligaments and cannot measure curvature, angles or ligament depth) of glenohumeral ligamentous insertions according to their pattern. Hereby, we present a Thiel-based anatomical study describing glenohumeral ligaments where our main aim is to describe and measure their glenoid (medial/proximal) insertions.

## 2. Materials and Methods

Thirty-nine shoulders were used in this study (Table 1, Table 2 and Table 3). Human tissue was obtained from volunteer body donors to the Applied Anatomy Laboratory at the Autonomous University of Madrid Department of Anatomy, Histology, and Neuroscience. Cadavers were subjected to transfemoral perfusion with 14.3 L of a water-based solution containing 3 g 3BO_3_, 30 mL ethylene glycol, 20 g NH_4_NO_3_, and 5 g KNO_3_, as well as 500 mL of another water-based solution consisting of 20 mL ethylene glycol and 1 mL 4-Cl 3-methyl phenol [15,16]. Exclusion criteria included post-mortem lapses longer than 48 h, a known history of shoulder pathology in clinical records at the time of cadaver reception, and presence of macroscopic lesions upon direct inspections of the shoulder joint. After complete separation of the upper limb from the trunk using a band saw, all vasculonervous structures of the axilla were sectioned and rebutted. The subscapular muscle was dissected from the subscapular fossa until its myotendinous insertion in the humeral head was visible. This junction was very carefully dissected, as its cephalic limit follows the same direction as the SGHL. After visualization from the frontal aspect of the shoulder joint capsule, shoulders were assigned to one of three possible patterns according to the amount of glenohumeral ligaments (Figure 1): pattern 1 (the ‘canonical’ one) when three glenohumeral ligaments were identified (SGHL, MGHL and AIGHL), pattern 2 when a single ligament was found, and pattern 3 when two ligaments were identified [4]. Afterwards, an LCD Vernier (Parkside®, Neckarsulm-Germany) digital caliper was used to measure the range of insertions of each possible ligament, both in the bone and in the labrum. Measurements were taken by a single observer (EGA) by laying (without compressing) the jaws of the caliper on the upper and lower ends of the coalescence of any given putative GHL and the glenoid surface, while ensuring the jaws were parallel to the glenoid rim by gently pushing the fixed step against it. A ligament was considered an independent entity when a macroscopic separation between fascicles was present at the site of insertion. Many GHLSs showed a bilaminar medial attachment, with labral insertions occupying a deeper plane than osseous insertions. Then, transections parallel to the glenoid surface were performed at the middle of the articular space, and measures were repeated. This was performed to correct the overlap between ligamentous insertions, which was present in some cases of pattern 1 and 3. This produces three different sets of data: a direct measurement of the glenoid insertion of any fascicle (measured directly over the anterior surface of the joint or after transection when overlaps between ligaments made the former measure unreliable, and reported in Table 1, Table 2, Table 3 and Table 4 with the suffix ‘o’ when related to a osseous insertion and ‘l’ when related to a labral insertion), a sum of the maximum length of insertions (reported in Table 1, Table 2, Table 3 and Table 4 as ‘Total’), and a sum of the maximum length of insertions that were apparent from an anterior view of the shoulder (reported in Table 1, Table 2, Table 3 and Table 4 as ‘Total Surface’); note that when ‘Total’ and ‘Total Surface’ do not yield the same result, some degree of overlap between superficial osseous and deep labral insertions was present). Data were analyzed using Microsoft Excel and IBM (Armonk, NY, USA) SPSS Statistics 19: Student’s T-test was employed to compare the data of canonical versus non-canonical patterns and to examine possible sex or laterality-related differences in morphometry. A Chi-square test was employed to test possible associations between laterality, sex and morphological pattern. Chi-square assumptions were assessed by examining expected cell counts. In analyses where more than 20% of cells had expected frequencies below 5, a Fisher test with Monte Carlo estimation was additionally performed. The level of significance was set at 0.05. Only proximal (scapular/glenoid) insertions were registered in this study.

## 3. Results

### 3.1. There Are Three Different Patterns of Glenohumeral Ligaments in the Anterior Aspect of the Shoulder

**Figure 2:**
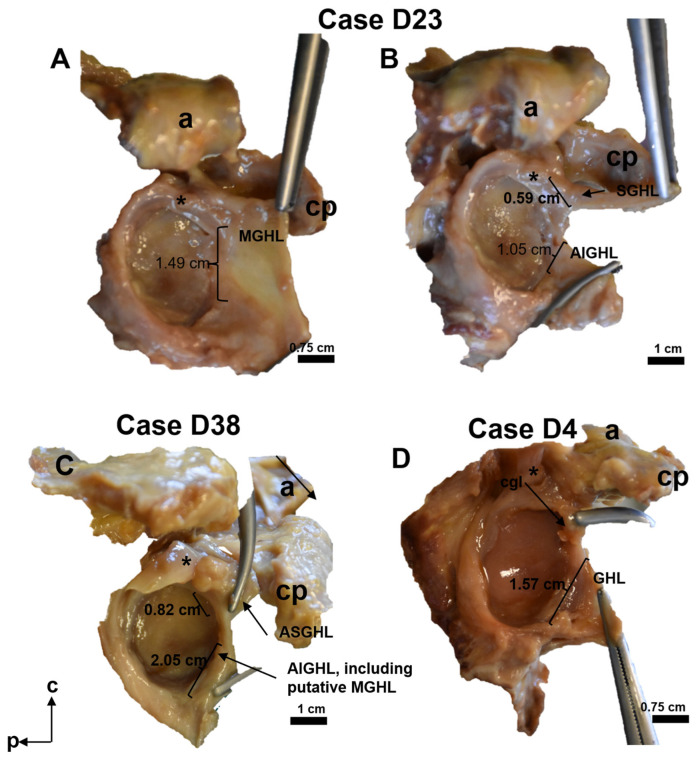
(**A**–**C**): Frontal views of the insertions of the glenohumeral ligaments at the level of the glenoid cavity, showing an example of a canonical pattern 1 (**A**,**B**) with the MGHL (**A**) located deeper to the SGHL and AIGHL due to its predominantly labral insertions. Picture C shows a rather atypical case of pattern 3 with two ligaments, a putative ASGHL and an amalgamated MGHL + AIGHL that barely reaches beyond the upper limit of 4 o’clock position in its most cephalic insertion. (**D**) shows a single GHL (probably a MGHL occupying the positions 3 to 5 o’clock). a: acromion; c: cephalic; cp: coracoid process; cgl: coracoglenoid ligament; p: posterior * represents an insertion of the long head of the biceps brachii and therefore the 12 o’clock position.

**Figure 3:**
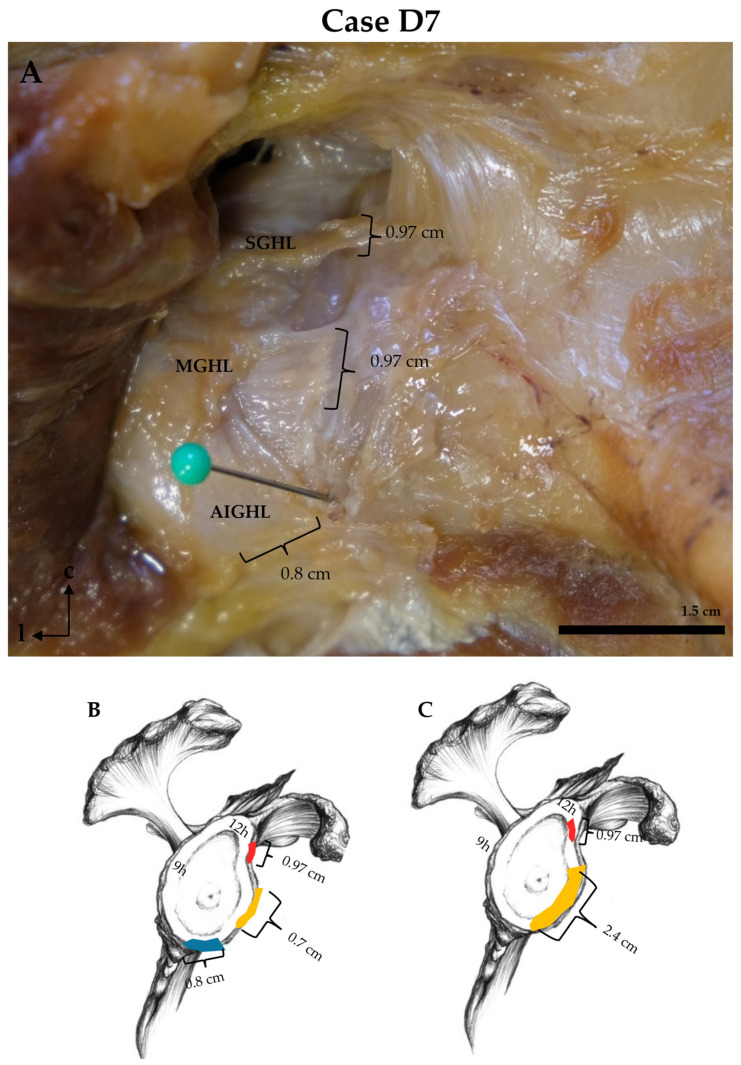
(**A**): canonical pattern with a markedly cephalized SGHL (12–1 o’clock and 0.97 cm) that shows both osseus (**B**) and labral insertions (**C**), and a wide MGHL with strong and wide osseous reinforcements (see (**B**)); its more caudal labral insertions are situated beneath the upper border of the AIGHL. See also Figure 1 for the color key.

**Figure 4:**
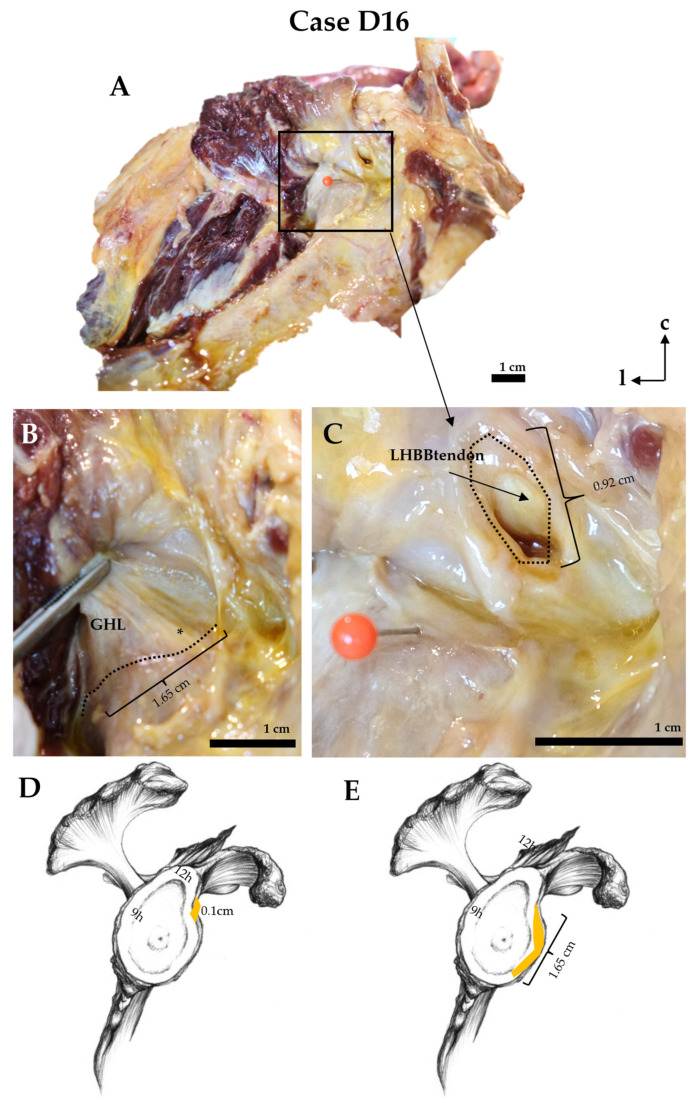
(**A**–**C**): A single ligament, GHL (**A**,**B**), covers the anterior surface of the shoulder joint capsule. It shows a wide labral insertion (2–5 o’clock and 1.63 cm, (**B**,**E**)) with additional osseous insertions (around 2 o’clock, (**B**) *, (**D**)), which may represent a caudally displaced SGHL, this displacement causing an exposure of the glenoid insertion of the long head of the biceps brachii (LHBB). This is shown in (**C**): a permeable, 0.92 cm wide foramen is defined around the tendinous insertion.

**Figure 5:**
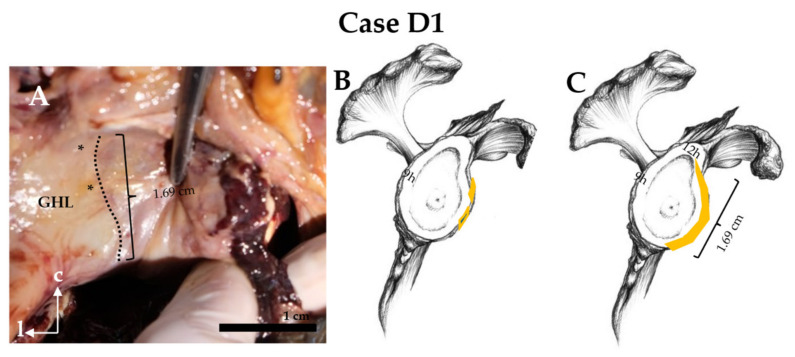
(**A**): A single ligament, GHL (**A**), covers the whole anterior surface of the shoulder joint capsule. It shows a wide labral insertion (2–6 o’clock and 1.69 cm, (**B**,**C**) *) with additional osseous insertions (around 2 and 3 o’clock, (**B**)). No permeable gateway to the articular space was found. Asterisks represent the positions of the osseous reinforcements.

**Figure 6:**
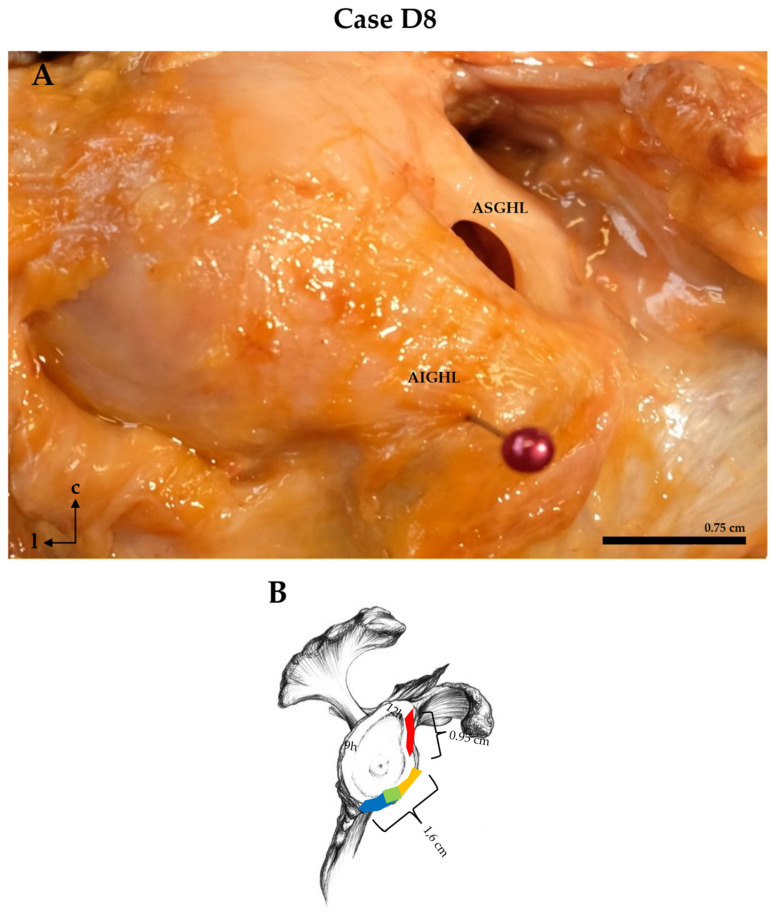
(**A**,**B**): An example of pattern 3: MGHL and IGHL seemingly conjoined as a single ligamentous band (AIGHL) with labral insertion of 1.6 cm (3–6 o’clock). The upper glenohumeral ligament is together with the coracohumeral and extends in the form of a band with almost apical (12–2 o’clock) insertions of 0.95 cm. There is a pin in the labral insertion of AIGHL. See Figure 1 for the color key.

Among the 39 cases studied, all of them showed at least one ligament (Figure 2, Figure 3, Figure 4, Figure 5 and Figure 6), almost invariably covering the area between 2 and 3 o’clock regarding its scapular insertions. Regarding the pattern, 30.8% of cases presented three ligaments (pattern 1, the ‘canonical’ one, Figure 1A, Figure 2A,B and Figure 3), 38.8% presented all GHLs amalgamated into one single ligamentous band (pattern 2, Figure 1B, Figure 2B, Figure 4 and Figure 5), and 30.8% presented two ligaments (pattern 3; Figure 1C, Figure 2C, Figure 3 and Figure 6). Morphometry is (Table 1, Table 2, Table 3 and Table 4) dependent on the pattern of insertion. Sex and laterality were unrelated to the patterning (χ^2^ = 1.17; sig. = 0.55 for sex and χ^2^ = 0.75; sig = 0.68/0.643 after Fisher’s exact test and Monte Carlo for laterality).

### 3.2. Pattern 1 Resembles the Canonical Description of the Glenohumeral Ligaments

In pattern 1 (Table 1), where the three ligaments are identifiable from a frontal view of the shoulder, 83% of SGHLs showed osseous insertions (range 0.28–1.58 cm; $\bar{x}$ = 0.69 ± 0.38), and 25% of cases showed labral insertions (range 0.41–0.98 cm; $\bar{x}$ = 0.66 ± 0.29). In this pattern, almost all (91.6%) of the MGHL showed pure labral insertions (range 0.56–2.4 cm; $\bar{x}$ = 1.2 ± 0.58), and osseous insertions represented slightly smaller fascicles that were mostly discrete reinforcements of the main body of the ligament, present in 25% of cases (range 0.55–1.49 cm; $\bar{x}$ = 1 ± 0.47). In AIGHLs, osseous insertions were present in 33.3% of cases (range 0.45–1.05 cm; $\bar{x}$ = 0.85 ± 0.26), and 75% of cases showed labral insertions (range 0.5–1.88 cm; $\bar{x}$ = 1.08 ± 0.49).

This table shows the classical arrangement of three fascicles within the glenohumeral ligament complex: superior, middle, and inferior, all of which are independently identifiable. SGHLo, MGHLo, and AIGHLo represent the osseous insertions of the glenohumeral ligaments (GHLs); SGHLl, MGHLl, and AIGHLl represent their labral insertions. L: left; R: right, PML: post-mortem lapse.

### 3.3. Pattern 2: A Single Glenohumeral Ligament in the Anterior Aspect of the Shoulder Joint

In pattern 2 (Table 2 and Figure 4), all ligaments coalesced in a single band, which is universally inserted in the labrum (range 1.57–3.6 cm; $\bar{x}$ = 2.19 ± 0.59); this band has osseous reinforcements in its upper border in up to half of the cases (53.3%) that probably represent remnants of a putative SGHL. These reinforcements range from thin connective bridges to true ligaments (range 0.1–2.09 cm; $\bar{x}$ = 1.056 ± 0.87).

This table illustrates the cases in which a single ligamentous fascicle is identified, corresponding to the middle fascicle of the glenohumeral complex. GHlo represents the osseous insertion of the middle glenohumeral ligament and GHLl its labral insertion.

### 3.4. Pattern 3: Two Ligaments Present in the Anterior Surface of the Shoulder

Pattern 3 (Table 3) is assigned when only two ligaments are identifiable from an anterior view of a dissected shoulder joint. In these cases, it is not possible to unambiguously define which ligament represents the superior, middle or anteroinferior one, so an anterosuperior GHL (ASGHL) and AIGHL were defined.The insertions of the ASGHL ranged between 0.4 and0.82 cm for osseous insertions ($\bar{x}$ = 0.6 ± 0.18) and between 0.46 and 2.16 for labral insertions ($\bar{x}$ = 0.8 ± 0.5). The insertions of the AIGHL in pattern 3 ranged between 1.18 and 2.5 cm for osseous insertions ($\bar{x}$ = 1.95 ± 0.5) and between 0.58 and 2.5 for labral insertions, excluding case D6 in which they were absent ($\bar{x}$ = 1.75 ± 0.576). Among all the shoulders classified as pattern 3, 75% showed a pattern that resembled a combination of a MGHL with a putative SGHL (see Figure 1C) (range 0.4–2.5 cm for osseous insertions and 0.42–2.5; for labral insertions) and the other 25% a combination of a MGHL + AIGHL or AIGHL + SGHL (range 0.47–2.05 cm for osseous insertions and 0.58–2.05 for labral insertions, see also Figure 1C). Based on its ‘clockface-defined’ insertions, no shoulder showed an absolute absence of ligaments inserted around 3 o’clock except case D38.

### 3.5. Clockwise Glenoid Insertions of the Glenohumeral Ligaments Related to Their Patterning

In 56% of cases, a GHL, either a SGHL in pattern 1 or an ASGHL in pattern 3, was inserted in the area immediately anterior to the supraglenoid tubercle, and its anterior aspect showed osseous insertions. In 12.8% of cases, this ligament exhibited exclusively labral insertions. In 10.2% of cases, a two-layered ligament was identified with both superficial osseous insertions and deep labral insertions.

All cases but one displayed a ligament with glenoid insertions covering the area around 3 o’clock and around the more anterior projection of the articular angle, which coincides with the ontological point of origin of the shoulder capsule (see Discussion); they were considered MGHL in pattern 1, single GHL in pattern 2 and part of either the ASGHL or AIGHL in pattern 3. Osseous insertions of these ‘central’ GHLs were present in 38.4% of cases, always in the anterior angle of the glenoid surface. Almost all cases were inserted in the labrum, meaning that this central fascicle was a somewhat deeper structure than the rest of the GHLs. In 30.7% of cases, it was a double-layered structure with both osseous and labral insertions encompassing almost its entire cephalocaudal length. In 7.69% of cases (see D1, D14L, D16 Table 1, Table 2 and Table 3), an almost purely labral central GHL was reinforced with a very thin cephalic fascicle of around 0.1 cm and inserted around 1 o’clock, probably corresponding with an amalgamated SGHL.

Around 46% of cases exhibited an AIGHL that was inserted in the osseous border of the anterior and inferior region of the glenoid (4 to 6 o’clock) around the lateral edge of the glenoid neck or in the immediately underlying labrum (38.4%). In 7.69% of cases, there was a close apposition between superficial osseous-inserted fascicles and deeper labral ones, forming a two-layered structure (see above), and no cases with a purely osseous insertion were identified, while overall, 23.07% of cases showed purely labral insertions. All results are recorded in Table 1, Table 2 and Table 3.

The patterning also influences the perimeter of ligamentous insertion as seen from the anterior surface of the joint (t = 2.131 and *p* = 0.04, comparing canonical pattern to non-canonical patterns). Sex and laterality did not show any statistically relevant relationship to the linear length of any set of insertions.

## 4. Discussion

This study is a descriptive study in soft-embalmed cadavers that provides morphometrical data for the scapular insertions of the glenohumeral ligaments, except the posterior ligaments; GHLs are essential for shoulder stability and are frequently subject to traumatic injuries. Quantitative studies on the glenohumeral ligaments are rare [2], typically based on few cases [3] or focused on only one ligament [17,18]. Also, a few studies [13,19] describe regional variability in a systematized way, although this description is usually performed from an intra-articular point of view. However, arthroscopic patterns largely coincide with gross anatomical ones [4]. To our knowledge, there are few quantitative studies of the glenohumeral ligaments as well as few shoulder studies in Thiel-embalmed cadavers (see also [20] for other studies using soft-embalming methods). In this work, we have not assessed the coracohumeral [21], posterior glenohumeral [3], and spiral [14] ligaments.

### 4.1. Technical Issues, Nomenclature, and Embryological Basis of Anatomical Variability

The main shortcomings of this study are those inherent to cadaveric-based studies. The fixation method, although allowing a good separation between structures in the connective tissue [4], hampers the histological assessment of the ligaments described and, therefore, a more precise identification. Additionally, complete information about subtle clinical details that may indicate shoulder pathology is not readily accessible to researchers. Degenerative changes of the capsule and labrum may alter the macroscopic differentiation of the glenohumeral ligamentous condensations, even in the case of absent obvious macroscopic changes. Prolonged reiterative use of the shoulder may also produce remodeling. In any case, our samples still belong to a real-world population, although inherently age-biased; the true native pattern of the shoulder joint (without degeneration and use-related remodeling) might only be present in newborn or early infant shoulders, and these samples are far beyond our reach. The dissection protocol offers the advantage of having previously separated the upper limb from the trunk, which frees up joint movements and eases the identification of the GHLs. This task is unfeasible when the limb hangs in a neutral position [21]. Also, as measures have been taken by a single observer, it is not possible to rule out inter-observer discrepancies. As this work is performed on cadaveric samples, it is also impossible to assure full morphometric coincidence in the clinical setting, where muscular tone, circulation, and physical activity may induce subtle changes in anatomic relations.

Nomenclature is also a relevant issue in this field. No precise classification of anatomical variants of GHLs has been established. As in previous publications by our group, a simple classification was employed based on the absolute number of ligaments visible in a frontal view of the shoulder [4]. This classification (and frequency data) matches the one developed for subscapular recesses [13,22,23], see also the work of Steinbeck et al., 1998 [19], which are (at least partially) the intra-articular counterpart of the cleavage planes between glenohumeral ligaments. However, this classification of patterns does not solve the problem of nomenclature. Thus, the presence of a single GHL (pattern 2) could be due to the disappearance of any pair of canonical ligaments. The presence of a pattern 3 could be due to the absence of any of the three canonical ligaments. Altogether, the findings reported here point to the necessity of reevaluating the nomenclature of ligaments in the anterior aspect of the shoulder joint. These ligaments are usually named according to a canonical pattern described in the 19th century [1], implying the existence of three ligaments [24] that are named ‘superior’, ‘middle’, and ‘inferior’, accordingly. Although the canonical pattern 1 is indeed persistent, more than half of the patients show alternative patterns from both the extraarticular and intraarticular points of view [4,13,22]. The existence of other patterns renders the present classification of glenohumeral ligaments potentially misleading, as the inconsistency between anatomical series suggests [2,13,17,19,22]. This also precludes accord between biomechanical studies.

**Figure 7:**
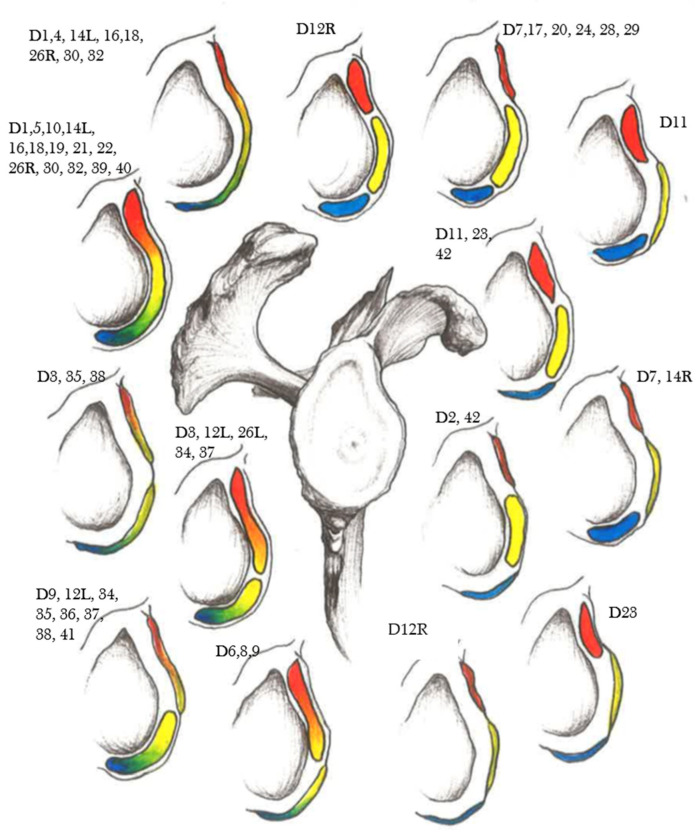
A schematic diagram showing the wide range of variability of glenoid insertions of the glenohumeral ligaments, depending on their pattern and the labral or osseous nature of their insertions, and the studied cases assigned to their corresponding morphological pattern. Note that some possible combinations are not present in our sample, yet plausible. Note also that, for the sake of clarity, the possible combinations when a given ligament can show both labral and osseous insertions are not shown, and therefore some specimens (e.g., D1, D14L, 23, 30, 32) are assigned to more than one combination. Color key is explained in Figure 1.

Overall, there are some issues regarding the histogenesis of the patterning and variability of the shoulder joint ligaments. The GHLs are altogether a combination of osseous and labral insertions that may or not superpose or be focally reinforced, and insertions may appear either cephalic regarding the glenoid surface (thus representing a SGHL), intermediate (MGHL) or caudal (AIGHL). This constellation of morphologies, partially represented in Figure 7, arises from the embryonic origin of the glenohumeral ligaments in the interzone of the developing joint [25]. At the end of the first trimester of gestation, the GHLs are seen as a osseous inserted band of connective tissue underlying the nascent subscapularis muscle (see Figure 5C in Hita-Contreras et al., 2018 [25]) with a thickening that coincides with the position of the putative MGHL, this being the first glenohumeral ligament to be identifiable, which may explain our finding of an almost universal presence of ligaments with insertions around the middle position (3 o’clock) of the anterior lip of the glenoid surface, independently of the insertion pattern. The wide range of insertions and the variability regarding their labral or osseous nature might be related to the distance between the thickening of the interzonal sheet of tissue that precedes the GHL and the interzonal thickening that precedes the labrum [25,26]. Therefore, the variability between patterns is explained by the variability in the cephalo-caudal position of the primitive interzonal thickening, and the variability between insertions is explained by its medio-lateral variability. Also, the primitive continuity of the interzonal thickening is probably the root of adult-life bridges between distinct ligaments, such as the frequent overlap between the most medial insertions of the SGHL/AIGHL and the lateral insertions of the coracoglenoid ligament, as seen in Figure 3A and in medical imaging [27].

### 4.2. Comparability to Other Studies

The main findings of this study are the morphometrical data for the scapular insertions of the glenohumeral ligaments, related to their pattern of insertion. Despite the high prevalence and cost of shoulder pathology, these kinds of quantitative studies are scarce and remain relatively inconspicuous [2]. Our morphometrical data largely match the more recent ones [3], although measures in this work are linear ones that do not consider the curvature/perimeter of the glenoid rim or the labrum. Also, we have not measured the footprint of the ligamentous insertion. However, the data presented here remain in a comparable range and consider patterns diverging from the canonical one, which were not recorded in the aforementioned study, probably due to its reduced sample size. Regarding the SGHL, our findings point in the same direction as other studies that try to reconcile long-standing discrepancies regarding the origins of this fascicle [17,28], which has been described to be inserted both in the supraglenoid tubercle and the superior labrum at the 1 o’clock position. This difference is likely related to the double-layered nature of the glenohumeral ligaments (see Results and Figure 7) and the fact that arthroscopic and gross anatomical studies describe different layers of the same structure [29]. According to the work of Kask and colleagues [29], oblique fibers of the superior glenohumeral ligament arise from the supraglenoid tubercle, and the direct fibers arise from the labral insertions. The results presentedhere reinforce the idea of two distinct fascicles of the SGHL and provide quantitative data for them. This can also be applied to the remaining GHLs, particularly to the MGHL, which is very frequently double-layered, showing almost invariably a deep, labral fascicle (Figure 7). Considering the patterning, around one third of the population shows only two anterior ligaments, matching the frequency described for MGHL agenesia [17], see also [22,30,31] insertions (see Material and Methods); however, there are very few cases of ligaments without insertions in the middle (3 o’clock) of the anterior lip of the glenoid cavity (2.5%). Remarkably, we have also not found any case of Buford complex [18,32,33]. Likely, the leaf-like MGHL reported in many studies corresponds to our pattern 2, and the cord-like MGHL corresponds to the canonical pattern 1 [31,34,35,36], at least in our sample. More complicated are the findings related to the insertions of the AIGHL. First, we are limiting our description to the ligaments that are identifiable on the anterior surface of the shoulder joint and not considering the PIGHL, which is probably functionally intermingled with the AIGHL. Second, in our sample, their range of insertion seems to encompass a much more caudal position than many studies [14,17,18], although other studies, including some recent ones, challenge these descriptions [3,37]. Again, discrepancies may be rooted in the method employed for defining the ligaments.

### 4.3. Clinical Considerations

The morphometric analysis of the insertions and the bilaminar structure (osseous + labral) of the glenohumeral ligaments is useful for understanding the stability patterns of the shoulder joint [38,39,40,41,42,43,44,45,46,47,48,49,50,51]. This should be proved with further biomechanical studies and clinical series, which areoutsidethe scope of this work.

All 39 specimens exhibited at least one ligamentous fascicle spanning the region between the 2 and 3 o’clock positions, reinforcing the idea that the anterosuperior glenoid represents a consistent site of capsular reinforcement [3,17]. The identification of a well-defined SGHL in more than half of the cases, predominantly with osseous insertions anterior to the supraglenoid tubercle, is consistent with previous descriptions linking this structure to stabilization of the long head of the biceps and control of anterior translation of the shoulder in external rotation [18]. However, the presence of an exclusively labral SGHL or a bilaminar configuration (observed in 12.8% and 10.2% of cases, respectively) may suggest a differentiated functional adaptation, potentially dependent on the ligamentous pattern and the degree of capsulolabral integration. This finding may explain why certain anterosuperior labral lesions are not necessarily associated with clinically significant instability [48,49].

The presence of non-canonical anatomical patterns (patterns 2 and 3) in more than 60% of the sample—either due to the absence of clear differentiation of the three ligaments or the presence of only two identifiable fascicles—suggests that the classical configuration described in anatomical atlases does not represent the norm but rather one of several possible variants. This finding aligns with some studies which propose that partial fusion of the GHLs may have clinical implications in entities such as the Buford complex, traditionally interpreted as anatomical variants but potentially associated with microinstability [3,49].Again, further clinical series are needed to confirm this hypothesis. Clinically, variability in the insertions of the MGHL and AIGHL may directly influence susceptibility to anterior or anteroinferior instability. In the present study, patterns with predominantly pure labral insertions showed a wider attachment range, which may correlate with increased capsular laxity and reduced anterior restraint. This association, while hypothetical, is consistent with biomechanical data [51]. Similarly, it is important to note that the anterior glenohumeral ligaments are frequently involved following trauma [38], leading, for example, to Bankart lesions (detachment of the anteroinferior labrum and glenohumeral capsule, typically involving the AIGHL), whose repair constitutes the primary treatment in cases of post-traumatic instability [39,40]. Other instability patterns may be related to injury of ligaments other than the AIGHL, particularly in the presence of non-canonical anatomical configurations (pattern 1) [41].

From a radiological perspective, differences between osseous and labral insertions are relevant for accurate interpretation of MRI and MR arthrography, which are essential for preoperative planning and its translation into surgical technique. Partial overlap of capsular structures may lead to false-positive diagnoses of labral detachment [18,42]. Therefore, recognition of anatomical variants, such as dual insertions or lack of ligament differentiation, should be part of the systematic preoperative radiological assessment, particularly in patients with recurrent instability or atypical findings at the anterior glenoid rim.

Furthermore, this duality of osseous and labral insertions may explain why lesions such as glenolabral articular disruption, which involve labral tears, do not always result in shoulder instability when osseous insertions remain intact [9,23,42]. In this context, the combined action of labral and osseous insertions may also account for the relative rarity of scapular avulsions of the GHLs compared to humeral avulsions [43] as well as for the subtle clinical presentation of ligamentous periosteal avulsions [44,45,46], which may lead to underdiagnosis. Conversely, insufficient repair (such as failure to address a potentially involved MGHL along with the AIGHL or incomplete restoration of all affected insertions) may result in recurrence of anterior shoulder instability following a single traumatic event [47]. Although this should be clinically confirmed, surgeon awareness about intrinsic variability of the structure may improve operatory outcomes. Also, the hypothesis that specific ligamentous configurations may modulate force vectors across the glenoid and inferior labrum is consistent with the observed distribution of SLAP and Bankart lesions in shoulders with morphological variations [52].

Finally, the observation that anatomical pattern was not significantly associated with sex or laterality may suggest a morphogenetic rather than adaptive component. However, the functional relationship between bilaminar insertions and dynamic shoulder stability warrants further research integrating morphometric data, high-resolution imaging, and postoperative clinical outcomes.

## 5. Conclusions

There are three different patterns of glenohumeral ligaments in the anterior aspect of the shoulder joint, the canonical pattern of a ‘superior’, plus ‘middle’ and ‘inferior’ ligaments represented in less than half of the cases.

All glenohumeral ligaments show distinct, and not necessarily overlapping, sets of labral and osseous insertions.

Altogether, we propose a nomenclature that defines a single GHL for pattern 2; a SGHL, MGHL and AIGHL for pattern 1; and an ASGHL and AIGHL for pattern 3.

## Figures and Tables

**Table 1:** Morphometric data of the glenohumeral ligaments corresponding to Pattern 1 (canonical). Measures are in centimeters.

Case	Sex	Age	PML	Cause	Laterality	SGHLo	SGHLl	MGHLo	MGHLl	IGHLo	IGHLl	Total	Total Surface
D2	M	87	12	Neoplasia	L	0.68			0.76	0.890		2.33	2.33
D7	F	73	33	MI	R	0.97		0.97	2.40		0.80	5.14	4.17
D11	F	98	29	COPD	R	0.63			1.57	1.030	1.03	4.26	3.23
D12	F	73	6	KF	R		0.41		0.60		1.63	2.64	2.64
D14	F	72	36	KF	R	0.40		0.55			0.89	1.84	1.84
D17	F	71	9	COPD	R	0.28			0.56		1.55	2.39	2.39
D20	F	64	6	Neoplasia	L	0.47			0.96		1.88	3.31	3.31
D23	M	65	6	Neoplasia	R		0.59	1.49	1.49	1.050		4.62	3.13
D24	F	78	26	NHL	R	0.52			1.85		0.93	3.30	3.30
D28	F	65	14	Neoplasia	R	1.58			1.48		0.50	3.56	3.56
D29	M	94	15	KF	L	0.45			0.89		0.54	1.88	1.88
D42	M	51	20	MI	R	0.98	0.98		0,88	0.450		3.29	2.31

**Table 2:** Morphometric data of the glenohumeral ligaments corresponding to Pattern 2 (single fascicle). Measures are in centimeters.

Case	Sex	Age	PML	Cause	Laterality	GHLo	GHLl	Total	Total Surface
D4	M	77	22	Neoplasia	L	1.57	1.57	1.57	1.57
D5	F	66	13	Neoplasia	R		1.73	1.73	1.73
D10	M	88	22	ReF	R		1.89	1.89	1.89
D14	F	72	36	KF	L	0.10	2.98	3.08	2.98
D16	M	79	9	Neoplasia	R	0.10	1.65	1.75	1.65
D18	F	80	15	MI (surgery)	R	2.07	2.07	4.14	2.07
D19	F	87	21	Pneumonia	R		3.60	3.60	3.60
D21	F	73	10	MI	L		2.61	2.61	2.61
D22	M	88	16	HF	R		1.96	1.96	1.96
D26	M	87	16	MI	R	0.94	2.80	3.74	2.80
D30	F	67	6	Neoplasia	R	2.09	2.09	4.18	2.09
D32	M	73	18	Neoplasia	L	1.48	2.30	3.78	2.30
D39	F	77	10	Neoplasia	L		2.24	2.24	2.24
D40	F	84	23	AD	R		2.50	2.50	2.50

**Table 3:** Morphometric data of the glenohumeral ligaments corresponding to Pattern 3 (two differentiated fascicles). Measures are in centimeters. In this pattern, two fascicles are observed, whose relative position with respect to the glenoid allows them to be classified as superior and inferior. ASGHLo, and AIGHLo represent the osseous insertions of the glenohumeral ligaments (GHLs); ASGHLl, and AIGHLLl represent their labral insertions.

Case	Sex	Age	PML	Cause	Laterality	ASGHLo	ASGHLl	AIGHLo	AIGHLl	Total	Total Surface
D3	F	51	24	Neoplasia	L	0.47	0.47	1.180	0.58	2.70	1.65
D6	M	82	7	Neoplasia	R		0.59	1.79	0.00	2.38	2.38
D8	F	74	39	COPD	R		0.95		1.60	2.55	2.55
D9	M	54	14	Neoplasia	R		0.46	2.50	2.50	5.46	2.16
D12	F	73	6	KF	L	0.42	0.42		1.46	2.30	1.88
D26	M	87	1	MI	L		2.16		1.00	3.16	3.16
D34	M	94	18	Sepsis	R	0.77	0.77		1.82	3.36	2.59
D35	M	77	12	Neoplasia	L	0.40		2.25	2.25	4.90	2.15
D36	F	71	40	Myeloma	L	0.70			2.14	2.84	3.04
D37	F	87	15	AD	R	0.46	1.06		1.72	3.24	2.78
D38	M	89	19	Pancreatitis	R	0.82		2.050	2.05	4.92	2.87
D41	M	84	17	COPD	R	0.77			2.12	2.89	3.49

**Table 4:** Summary of morphometric data in a pattern-wise format.

Pattern	Ligament	InsertionType	*n* (%)	Range (cm)	Mean ± SD (cm)
**Pattern 1**	SGHL	Osseous	10/12 (83.3%)	0.28–1.58	0.69 ± 0.38
	SGHL	Labral	3/12 (25.0%)	0.41–0.98	0.66 ± 0.29
	MGHL	Osseous	3/12 (25.0%)	0.55–1.49	1.00 ± 0.47
	MGHL	Labral	11/12 (91.6%)	0.56–2.40	1.20 ± 0.58
	AIGHL	Osseous	4/12 (33.3%)	0.45–1.05	0.85 ± 0.26
	AIGHL	Labral	9/12 (75.0%)	0.50–1.88	1.08 ± 0.49
**Pattern 2**	Single GHL	Osseous	8/15 (53.3%)	0.10–2.09	1.06 ± 0.87
	Single GHL	Labral	15/15 (100%)	1.57–3.60	2.19 ± 0.59
**Pattern 3**	ASGHL	Osseous	7/12 (58.3%)	0.40–0.82	0.60 ± 0.18
	ASGHL	Labral	10/12 (83.3%)	0.46–2.16	0.80 ± 0.50
	AIGHL	Osseous	5/12 (41.7%)	1.18–2.50	1.95 ± 0.50
	AIGHL	Labral	11/12 (91.6%)	0.58–2.50	1.75 ± 0.58

## Data Availability

All data, including detailed photographical records of the dissections, are fully available on request to EGA or IPS. Most of the cadaveric material is still stored although not completely preserved as further work has been developed onthe same specimens.

## Abbreviations

The following abbreviations are used in this manuscript:

GHL/GHLs	Glenohumeral ligament/s
AIGHL	Anteroinferior inferior glenohumeral ligament
ASGHL	Anterosuperior glenohumeral ligament
MGHL	Middle glenohumeral ligament
SGHL	Superior glenohumeral ligament
PIGHL	Posterior glenohumeral ligament
CHL	Coracohumeral ligament
CGL	Coracoglenoid ligament
LHBB	Long head of the biceps brachii
